# The validation of biomarkers of metabolic efficacy in infant nutrition

**DOI:** 10.1111/nbu.12341

**Published:** 2018-08-10

**Authors:** S. Furse, L. Richardson, A. Koulman

**Affiliations:** ^1^ University of Cambridge Cambridge UK

**Keywords:** biomarkers, infant nutrition, lipid, mass spectrometry, triglyceride, validation

## Abstract

Breastfeeding is regarded as the ideal way to nourish infants. However, feeding with formula milk is also common in much of the West. Despite this, the function of the molecular components of breast and formula milks are not fully understood, less still the relationship between the composition of the milk and the infant's metabolism and how this influences the infant's development. The Biotechnology and Biological Sciences Research Council‐funded project ‘The validation of biomarkers of metabolic efficacy in infant nutrition’ aims to identify lipid biomarkers that can be used to study the effect of diet on growth and development of infants. In this work, we have been able to validate these markers. Here, we present an approach to biomarker discovery that has new depth and will inform research questions about how metabolism is governed, and which species can be used to identify situations where metabolism is becoming defective.

## Introduction

The national and international guidelines for infant nutrition recommend breastfeeding (WHO 2007, 2009) and it is widely regarded as an important part of the best start to a baby's life. This consensus has led to the recommendation that babies are breastfed exclusively for the first 6 months, followed by a transitional period of a year or more to establish feeding on solid foods as currently advised by the National Health Service (NHS) in the UK (NHS Choices 2018). However, it is not always possible to breastfeed exclusively, and so formula milk and other sources of nutrition are used. This variety of feeding regimes and the evidence for considerable malnutrition in infants in some areas (IFPRI 2016), coupled with the variety of growth and development trajectories of children, suggests that there is much still to learn about the relationship between feeding and infant development. The traditional approach has been to determine the total lipid and fat concentration in milk and track the children's development. However, the mixture of lipids and fats in the diet of the infant, combined with endogenous biosynthesis of lipids and fats, leads to a complicated profile of lipids in the circulation of the infant. Furthermore, these molecular species have a role in growth and development. A lack of understanding about how the lipid and fat composition of the diet affects infant development also limits understanding of the effect of the maternal diet and lifestyle on these processes. The detailed analysis of the circulating lipid profile of the infant, using lipid profiling techniques, allows investigation into how diet influences the circulating lipids and whether these are associated with the infants’ growth and development. Lipid profiling, also known as lipidomics, typically uses mass spectrometry to measure the concentration of all detectable lipids in a sample simultaneously. This technique can yield information on several hundred species and relies on advanced data processing and analysis to determine how lipid profiles differ between, for example, breastfed and formula‐fed infants (Koulman *et al*. 2014).

The lipids and fats in the diets of infants early in life are of particular importance as they provide a rich source of energy and raw materials, such as membrane components, indicating that they have a crucial role in supporting metabolic activity of this period. Current understanding of the lipid and fat profile of milk is basic and typically focused on fatty acids and total triglyceride levels (Pons *et al*. 2000; Mitoulas *et al*. 2002, 2003; Morera *et al*. 2003; Mandel *et al*. 2005; McAnoy *et al*. 2005; Lísa *et al*. 2009; Moltó‐Puigmartí *et al*. 2011; Linderborg *et al*. 2014). It is not clear how the profile of triglycerides changes with diet, ethnicity or through the period of lactation. Relatively little is known about the phospholipid fraction, particularly the anionic lipids. Furthermore, there is little information on the sterols in human breastmilk. It is, therefore, not known whether certain species are essential for healthy growth or are only associated with poor growth.

In order to investigate this, a comprehensive profiling of fats, lipids and sterols is required. This approach requires the analysis of a large number of possible molecular species (we profile around up to 4000). However, only a handful of these may be significant in directing the trajectory of an infant's development. At the same time, due to obvious ethical reasons, blood sampling will often be limited to heel pricks, yielding dried blood spots, which limit the number of lipids that can be measured robustly. It is therefore the aim of the Biotechnology and Biological Sciences Research Council (BBSRC)‐funded project ‘The validation of biomarkers of metabolic efficacy in infant nutrition’ to develop an assay capable of identifying and quantifying those lipids that can be used as biomarkers. In addition, the project aims to demonstrate which lipid and triglyceride species are important in relation to infant nutrition, growth and development; this relies upon proper validation of those biomarkers. Validation in this context refers to using a second cohort in order to check the results (candidate biomarkers) of the first, and their relationship with the effect(s) of interest.

## Strategy for using validation in metabolic studies

Generally, omics‐based studies adopt the approach of measuring very many components in small sample numbers. Many research groups find large sample numbers experimentally challenging in omics‐based experiments. The imbalance between sample numbers and analyte numbers can lead to the identification of associations without a clear understanding of whether these relationships are robust. In the last decade, it has emerged that not all the associations identified in omics studies could be repeated and for the majority there have been no attempts to repeat or validate the discoveries. This is partly due to the prohibitive cost of omics‐based studies. Moreover, only a few strategies for validation have been suggested, few of which have themselves been validated, suggesting that a ‘go‐to’ structure for establishing repeatability does not yet exist.

Validation of biomarkers requires that a precise relationship between the presence or abundance of that biomarker and the observed effect is evident. The first step is to embark on a pilot study. This set of samples forms the discovery samples set, they are the samples used for the discovery of candidate biomarkers. This discovery set (pilot study) needs to be selected carefully in order to contain sufficient participants for the effects to be seen clearly. This approach is aimed at highlighting proto‐candidate biomarkers. Statistical approaches are used on the results from the discovery set to identify species whose abundance differs (candidate biomarkers) and correlate (either proportionally or inversely) with the effect.

The approach we favour builds on this first step by using it to inform the analysis of a second cohort that is distinct from the first but comparable to it. For example, a study of a cohort of breastfeeding‐only mother and baby pairs from The Gambia might be followed up by a cohort of breastfeeding‐only mother and baby pairs from South Africa. Another example would be a study of a cohort of mixed‐fed babies born to Caucasian parents in Cambridge that might be followed up by a cohort with a similar feeding pattern from Denmark.

This ‘dual‐cohort’ approach offers the means for identifying biomarkers and includes the element of repeating measurements, but in a way that offers an objective test. This provides a solution to concerns raised about repeatability and thoroughness in pre‐clinical research (Macleod 2011; Yong 2012; Russell 2013), while also being scientifically informative. The dual‐cohort approach is more challenging for the discovery of biomarkers, as it is mostly based on studies with healthy participants that are not under strict metabolic control and are free‐living. This will mean considerable variation between the samples. Thus, where results indicate the presence of candidate biomarkers consistently, they are therefore likely to be most robust.

## Early results of the ‘validation of biomarkers of metabolic efficacy in infant nutrition’ project

The first project we carried out using a dual‐cohort approach was aimed at the discovery of robust lipid biomarkers for the relationship between infant lipid metabolism and type of milk feeding – either fully breastfed, fully formula‐fed or receiving a mixture of both formula and breastmilk. We have developed a robust method to determine the circulating lipid composition of infants using dried blood spot samples (Koulman *et al*. 2014). This method was applied to samples from the *Cambridge Baby Growth Study* (Petry *et al*. 2007; Lu *et al*. 2016) and we found that a limited number of circulating lipids were able to distinguish breastfed from formula‐fed infants at 3 months *post‐partum* (Prentice *et al*. 2015). We followed these findings up in two smaller cohort studies that would give us the opportunity to determine whether these lipids were indeed biomarkers of infant nutrition. From this work, we were able to identify and validate three lipids: a phosphatidylcholine (PC) with two fatty acid residues that comprise 35 carbons and two double bonds [PC(35:2)] and two sphingomyelins (SM) SM(36:2) and SM(39:1) that could be used collectively as biomarkers for infant nutrition during early development. Through the validation we were able to show that these biomarkers can be used to determine whether young infants (3–6 months) are breastfed or receive formula milk, and even whether an infant is fed a mixture of both (Acharjee *et al*. 2017).

We have also adopted the dual‐cohort approach in our work on identifying biomarkers for infant growth. We published evidence that the presence of several lipid and triglyceride isoforms have a relationship with baby weight (Prentice *et al*. 2015). PC(18:1/16:0) and PC‐O(34:1) correlated with weight gain, where PC(20:4/18:0), PC‐O(36:4), PC(36:4) and SM(d18:2/16:0) were inversely proportional to weight gain (Koulman *et al*. 2014; Prentice *et al*. 2015). In order to validate these candidate biomarkers formally, we have used a cohort of mother‐and‐baby pairs, this time from The Gambia. This cohort differs from the first in that it comprises only exclusively breastfed infants, has a different ethnicity profile and samples are drawn from a different region. This means that the mothers have different diet and living conditions. Furthermore, sample collection methods differed (the first study used dried blood and milk spots, the second fresh plasma and milk samples). Early results from the Gambian cohort indicate that the measurements taken in the first set are repeatable. The abundance of cholesterol and cholesteryl esters is also similar, as is the isoform profiles of PCs and triglycerides.

Our dual‐cohort approach differs from a like‐for‐like repetition that has been advocated by psychologists (Yong 2012). The latter method undoubtedly has merits, but we argue that our approach is scientifically relevant and ideal for the type of hypothesis we wish to test. First, our approach indicates that the effects we observe are not an artefact of the sampling methods used, or the ethnicities of the cohort. Second, the geographically distinct cohorts widen our understanding of the practise of breastfeeding, allowing subtler effects to be observed and thus widening the breadth of the study. Third, it informs the magnitude of evidence for or against general effects. For example, data from both of the cohorts described *vide supra* broadly support the conclusion that *de novo* lipid and triglyceride biosynthesis in mammary glands is considerable (Hansen *et al*. 1984; McManaman 2009; Mohammad *et al*. 2014).

## Future perspectives

Current evidence suggests that the dual‐cohort approach is fit‐for‐purpose for the validation of candidate biomarkers. The approach is broad as the markers identified could be for dietary intake, nutritional status as well as for health, disease or development. It also provides an approach that a variety of researchers can use to assess and validate candidate biomarkers, in the design of omics‐based projects. The concept of the dual‐cohort approach may also find use outside of life sciences research where validation is required, such as studies in sociology and psychology, and may also be used in physical sciences to explore effects observed in a number of independent systems.

However, further work is required to make it possible to apply this approach to any other study. For example, it is important to understand which parameters can be changed and by how much. There is a clear trade‐off between the risk of finding no effect – which can miss possible biomarkers – and the need for more work to validate the markers identified. The scientific value is in producing evidence that demonstrates that a given effect is (surprisingly) independent of parameters that seem relevant on paper. This may not be predictable in advance. For example, a trivial‐sounding procedure, such as sample handling and lipid extraction, may require more care than expected (Furse *et al*. 2015).

In order to explore the relationship between feeding and infant development, maternal and infant plasma samples are required, along with breastmilk and faecal samples. All these samples must be handled in a way that is sympathetic to the presence of factors that damage the lipid fraction or may bias it. For example, plasma samples tend not to contain bacterial cells, which can both produce and destroy lipids, though faecal samples will. However, plasma samples may be richer in lipases that remain active *ex vivo*, thus altering the lipid profile of the sample after collection. Approaches to sample handling and processing differ considerably between research groups and studies, too. Meta‐analyses indicate that this can have a considerable impact on the relative and absolute abundance of lipids that is recorded and published (Furse *et al*. 2015). A similar effect has been observed in lipid isolation procedures, leading to the development of new methods (Furse *et al*. 2017). This hints that there are features of studies that cannot be altered if they are to be comparable, and thorough validation of methods used is necessary to minimise the risk of this.

We, therefore, suggest that the dual‐cohort approach represents a good testing structure that can be tailored for the needs of individual scientific questions. In the context of infant nutrition, this approach is able to identify candidate biomarkers that may be involved in shaping infant growth. This may be important in developing our understanding of how infant growth is governed and informing feeding regimes for infants who cannot or should not be breastfed as well as the relationship between feeding and development in healthy individuals.
